# Supplementary material for: Chemical controls on iron distributions across the subsurface South Pacific Ocean
Source: Nat Commun. 2026 Apr 15;17:3533. doi: 10.1038/s41467-026-72070-y (PMC13087175; doi:10.1038/s41467-026-72070-y)
Supplement: Supplementary file 1 — Supplementary Information [file 41467_2026_72070_MOESM1_ESM.pdf]

Chemical controls on iron distributions across the subsurface South Pacific Ocean

M. Gledhill*[1], K. Gosnell[1], M. P. Humphreys[2], L. Delaigue[2,3], N. Helle[1], K. Zhu[1], P. Lodeiro[4], C. Rey-Castro[4], E.P. Achterberg[1]

1. GEOMAR Helmholtz Centre for Ocean Research Kiel, Kiel, Germany

2. NIOZ Royal Netherlands Institute for Sea Research, Department of Ocean Systems, Texel, the Netherlands

3. Sorbonne Université, CNRS, Laboratoire d'Océanographie de Villefranche, Villefranche-Sur-Mer, France

4. Department of Chemistry, Physics and Environmental and Soil Sciences, University of Lleida and AGROTECNIO-CERCA Centre, Lleida, Spain

*corresponding author: mgledhill@geomar.de

Supplementary Methods.

Chemical equilibrium calculations for simultaneous prediction of multiple Fe species

Speciation calculations in ORCHESTRA (version 7, 2022, downloaded from https://orchestra.meeussen.nl/)[1] were determined by ion-pairing using equilibrium constants[2] with activities corrected for ionic strength using the Davies model[1] and thermodynamic stability constants based on those of minteq.v4[3]. The non-ideal competitive interaction (NICA)-Donnan model represents heterogeneity of complex mixtures of binding sites that occur in natural systems via a bimodal distribution of binding sites[4] (Eq. 2). The NICA component of the model is based on the Sips/Langmuir-Freundlich isotherm which is a modified version of the Langmuir isotherm[5]. The Langmuir isotherm is a fundamental model for surface adsorption processes[6] often applied in marine systems to calculate conditional stability constants and ligand concentrations[7]. The Langmuir isotherm is suitable for description of interactions between one specific ligand and a metal. Application of the Langmuir isotherm to complex systems results in parameters that are difficult to interpret[5,8]. The Sips/Langmuir-Freundlich isotherm modifies Langmuir to account for interactions between a metal and multiple binding sites (ligands), where the properties of the individual binding sites cannot be determined because of the complexity of the system. The Donnan part of the model accounts for electrostatic interactions[9,10]. In the NICA model, the total availability of cation binding sites is set by the amount of proton binding sites ($Q_{\mathrm{maxH,1}}$ and $Q_{\mathrm{maxH,2}}$) and the width of each binding mode (p). Parameters describe total amount bound (Q_j) of component j (e.g. Fe, calcium, magnesium etc) present at concentration c_j with median affinities ($\bar{K}_{j,1}$ and $\bar{K}_{j,2}$). The "non-ideality", which is related to stoichiometry[11], is described by the parameters $n_{j,1}$ and $n_{j,2}$.

$$Q_j = \frac{n_{j,1}}{n_{H,1}} \cdot Q_{\mathrm{maxH,1}} \frac{(\bar{K}_{j,1} \cdot c_j)^{n_{j,1}}}{\sum_j (\bar{K}_{j,1} \cdot c_j)^{n_{j,1}}} \times \frac{\left[\sum_j (\bar{K}_{j,1} \cdot c_j)^{n_{j,1}}\right]^{p1}}{1+\left[\sum_j (\bar{K}_{j,1} \cdot c_j)^{n_{j,1}}\right]^{p1}} +$$

$$\frac{n_{j,2}}{n_{H,2}} \cdot Q_{\mathrm{maxH,2}} \frac{(\bar{K}_{j,2} \cdot c_j)^{n_{j,2}}}{\sum_j (\bar{K}_{j,2} \cdot c_j)^{n_{j,2}}} \times \frac{\left[\sum_j (\bar{K}_{j,2} \cdot c_j)^{n_{j,2}}\right]^{p2}}{1+\left[\sum_j (\bar{K}_{j,2} \cdot c_j)^{n_{j,2}}\right]^{p2}} \quad (1)$$

Calculations of weighted mean effective iron affinities for occupied DOM and POM binding sites.

Effective Fe affinities for occupied DOM and POM binding sites were calculated from the concentration of Fe present in the Donnan phase (i.e. "within" the permeable organic molecule or particle) following an analytical solution[12]. The analytical solution was implemented in R and is available online.

Calculation of Fe′ using conditional stability constants and ligand concentrations.

The binding of iron to ligands is described by the mean conditional affinity coefficients ($\overline{K'_{FeL}}$). A 1:1 stoichiometry is assumed, and $\overline{K'_{FeL_i}}$ for a ligand group L_i is defined as

$$\overline{K'_{FeL_i}} = \frac{c_{FeL,i}}{c_{Fe'} \times c_{L',i}} \tag{2}$$

Where c_{FeL} is the concentration of the Iron complex, and $c_{Fe'}$ and $c_{L',i}$ are the concentrations of unchelated Fe and ligand, respectively. Since the total number of ligands is much greater than 2, then $\overline{K'_{FeL_i}}$ is a mean affinity of the ligands that can be observed (by titration) under the given conditions of the experiment, and c_{Li} is the total concentration of the all the ligands that contribute to the determined $\overline{K'_{FeL_i}}$. The number of ligands detected is determined by the confidence with which multiple parameters can be fitted to titration data[13,14] and determined parameters (i.e. c_{Li} and $\overline{K'_{FeL_i}}$) are conditional to the experimental pH used for parameter derivation (usually pH 8), ionic strength, temperature and the ambient dissolved iron concentration[15]. Here, we estimate $c_{Fe'}$ from c_{Li} and $\overline{K'_{FeL_i}}$ using data obtained on a previous GEOTRACES transect across the SPO at 16°S[16].

For a two-ligand system, inorganic iron concentrations are derived from $\overline{K'_{FeL_i}}$ and c_{Li} using the following polynomial expressions. The analytical solution, which we undertook using the polyroot function in R, produces one real number[17,18]

For one ligand the expression is

$$0 = {c_{Fe'}}^2 + A_1 \cdot c_{Fe'} - B_1 \tag{3}$$

where

$$A_1 = c_L + \frac{1}{\overline{K'_{FeL_i}}} - c_{DFe} \tag{4}$$

and

$$B_1 = \frac{c_{DFe}}{\overline{K'_{FeL_i}}}. \tag{5}$$

For two ligands the expression is

$$0 = {c_{Fe'}}^3 + A_2 \cdot {c_{Fe'}}^2 + B_2 \cdot c_{Fe'} - C_2 \tag{6}$$

where

$$A_2 = c_{L1} + c_{L2} + \frac{1}{\overline{K'_{FeL_1}}} + \frac{1}{\overline{K'_{FeL_2}}} - c_{DFe} \tag{7}$$

and

$$B_2 = \frac{c_{L1}}{\overline{K'_{FeL_2}}} + \frac{c_{L2}}{\overline{K'_{FeL_1}}} + \frac{1}{\overline{K'_{FeL_1} \cdot K'_{FeL_2}}} - c_{DFe} \cdot \left(\frac{1}{\overline{K'_{FeL_1}}} + \frac{1}{\overline{K'_{FeL_2}}}\right) \tag{8}$$

and

$$C_2 = \frac{c_{DFe}}{\overline{K'_{FeL_1} \cdot K'_{FeL_2}}}. \tag{9}$$

Supplementary Table

Table S1. Physical and chemical parameters used for eOMPA and results of linear regressions between DFe concentration and percentage water mass composition for each water mass identified across the SPO. Results of a regression analysis of DFe as a function of water mass composition (r^2, significance (p value) and number of observations) are shown. The analysis did not include the station at 117°W over the EPR or at 177°E (close to the Monowai Volcano) since they were strongly influenced by Fe sources. Only values at depths >250 were considered in the analysis. ESSW: Equatorial SubSurface Water AAIW: Antarctic Intermediate Water, UCDW: Upper Circumpolar Deep Water, LCDW: Lower Circumpolar Deep Water, NPDW: North Pacific Deep Water.

		ESSW	AAIW	UCDW	PDW	LCDW
Temperature °C		9	5.55	2.44	1.82	1.6
Salinity		34.6	34.25	34.58	34.64	34.71
Oxygen (µmol kg^{-1})		13.6	250	162	132	200
Phosphate (µmol kg^{-1})		2.97	1.75	2.4	2.72	2.2
Total nitrate + nitrite (µmol kg^{-1})		32.7	25.3	34.5	38.2	32.4
Silicate (µmol kg^{-1})		29.81	13	83	150	116
Density (kg m^{-3})		1026.5	1027	1027.6	1027.7	1027.8
% water mass ~ DFe	r^2	0.010	0.02	0.003	0.007	0.003
	p-value	0.73	0.024	0.40	0.08	0.39
	n	17	266	199	431	260

Supplementary Figures

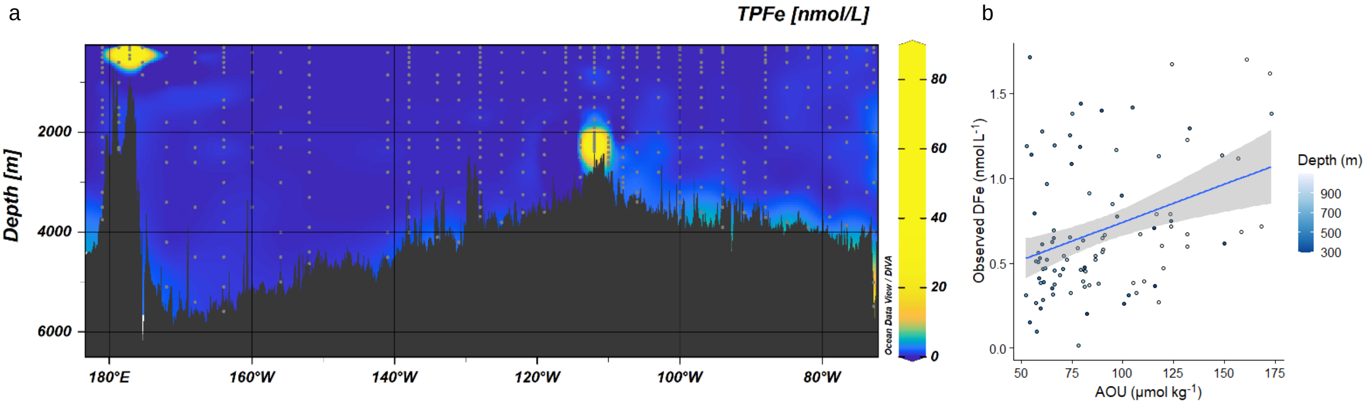

Supplementary Fig. 1a Observed distribution of total particulate iron (TPFe) across the South Pacific. Note non-linear scale of colour bar. **b** Plot showing relationship between observed DFe concentrations and apparent oxygen utilization (AOU) at depths between 300 and 1000 m, where Antarctic Intermediate Waters comprised the dominant water mass. Line shows linear correlation, with grey shaded area equal to ±1 standard error.

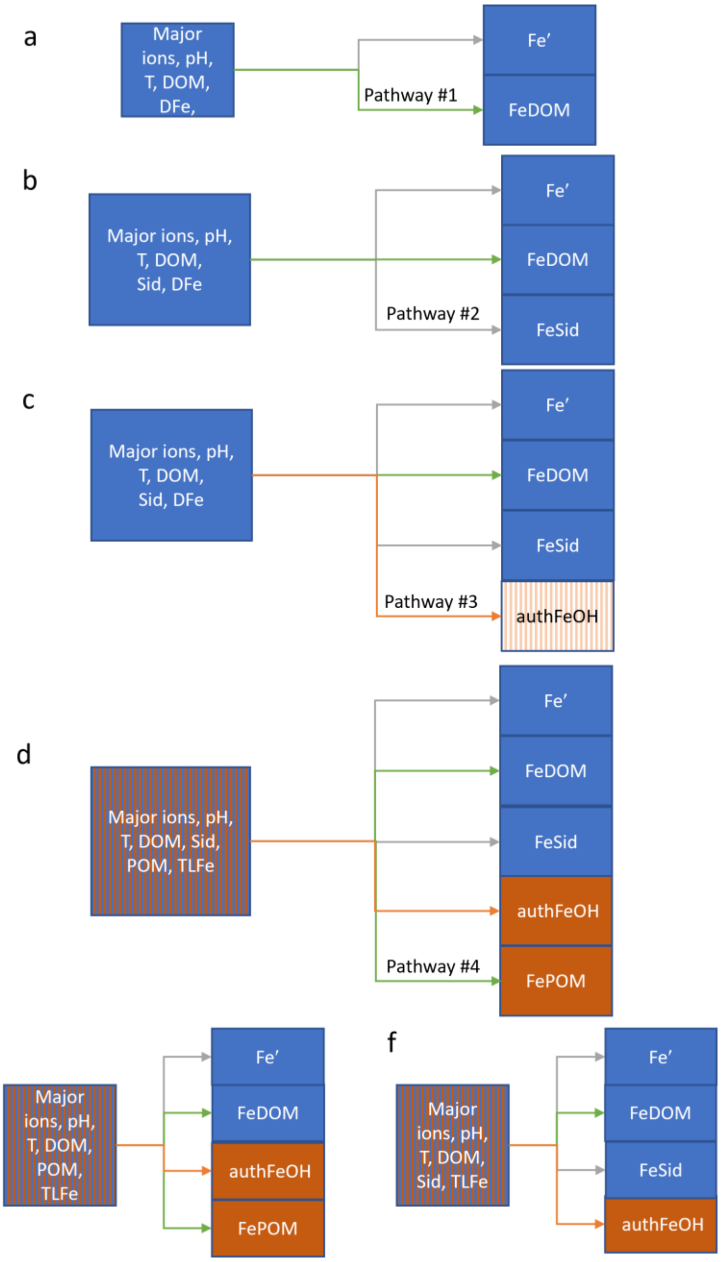

Supplementary Fig. 2. Overview schematic for coupled speciation calculations undertaken in ORCHESTRA[1]. **a-c** Pathways #1-#3 were evaluated using the observed concentration of DFe (DFe_{obs}) to constrain Fe speciation between dissolved pools (blue boxes: Fe′, FeDOM and FeSid). In pathway #3, authFeOH (hashed orange box) could be in the process of precipitating from Fe′, or already present as colloidal Fe (<0.2 µm). **d** Pathway #4 additionally incorporates Fe binding to POM and uses Total Labile Fe concentrations ($TLFe_{obs}$) as the Fe input ($TLFe_{obs} = DFe_{obs} + LPFe_{obs}$). Predicted DFe ($DFe_{pred}$) was then calculated from the sum of the concentrations of Fe′, FeSid and FeDOM (blue coloured boxes), whilst $LPFe_{pred}$ was calculated from the sum of authFeOH and FePOM (orange boxes). We note that although we allocate all predicted authFeOH to the particulate phase, some could be present as colloidal Fe (between 0.02 and 0.2 µm). **e-f** We made two further model calculations based on the schematic shown in **d** to examine the sensitivity of results to the absence of **e** pathway #2 (Sid) or **f** pathway #4 (POM). Connector colour indicates the method used to parameterize the pathways. Grey: equilibrium constants, green: NICA-Donnan model and orange: $FeOH_3(s)$ precipitation.

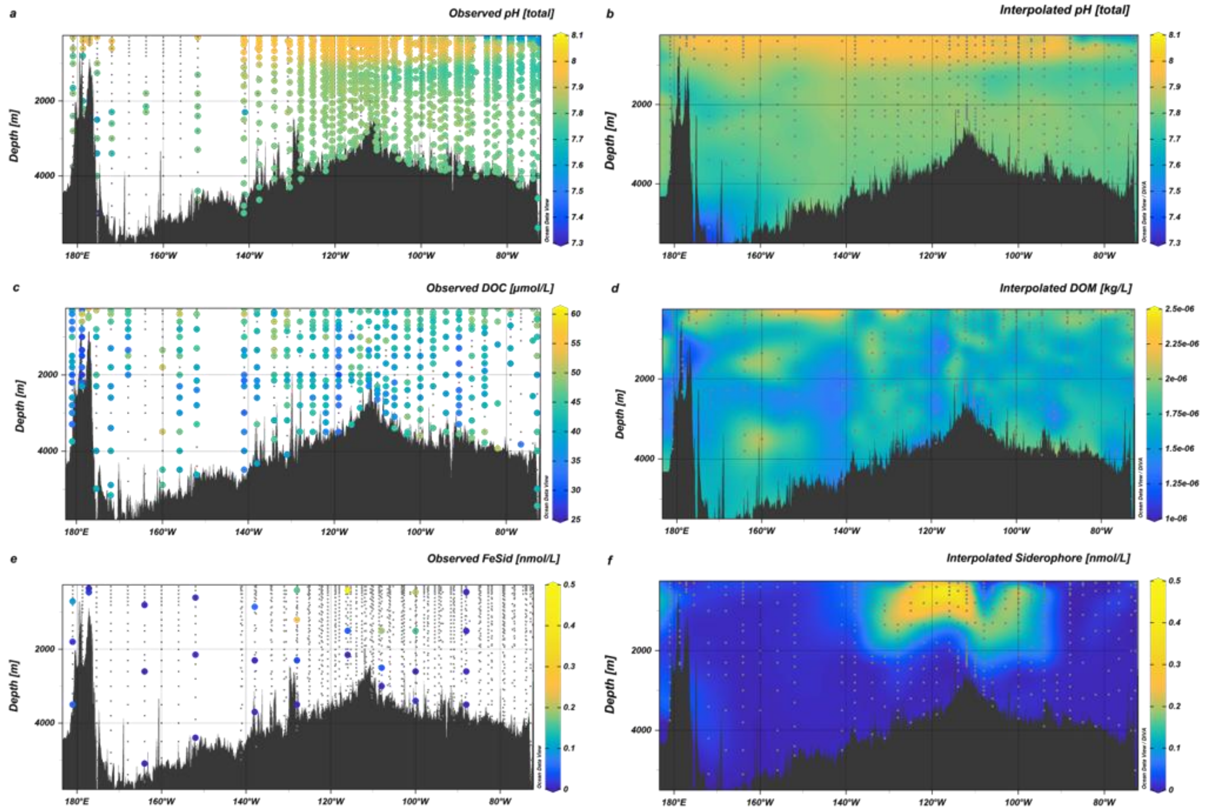

Supplementary Fig. 3. Distributions of observed (a, c, e) values and resultant interpolated (b, d, f) values used for model input. **a** and **b** show pH (shown here on the total scale, but adjusted to the NBS scale for the calculations), **c** observed dissolved organic carbon (DOC) and **d** interpolated dissolved organic matter (DOM), **e** observed Fe bound siderophore concentration and **f** interpolated siderophore concentration. Only depths below 250 m are considered in this study. In **a**, pH values east of 146°W are taken from GLODAPv2022[19], whilst those west of 146°W were determined in this study. Grey points in **b**, **d** and **f** show depths at which calculations were undertaken.

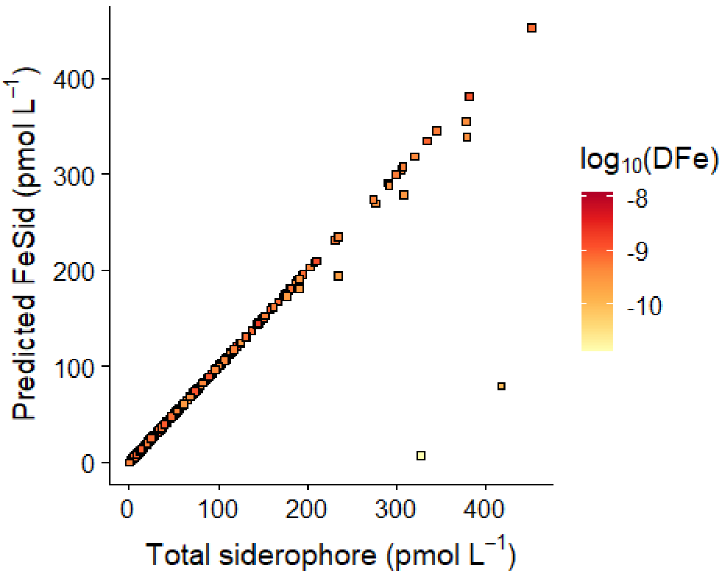

Supplementary Fig. 4. Relationship between the interpolated total siderophore concentration (model input) and Fe bound to siderophores predicted from the model (FeSid). Colour bar shows $\log_{10}$ DFe concentrations (mol L^{-1}). The relationship is not always directly linear as DOM can compete with siderophores for Fe[20].

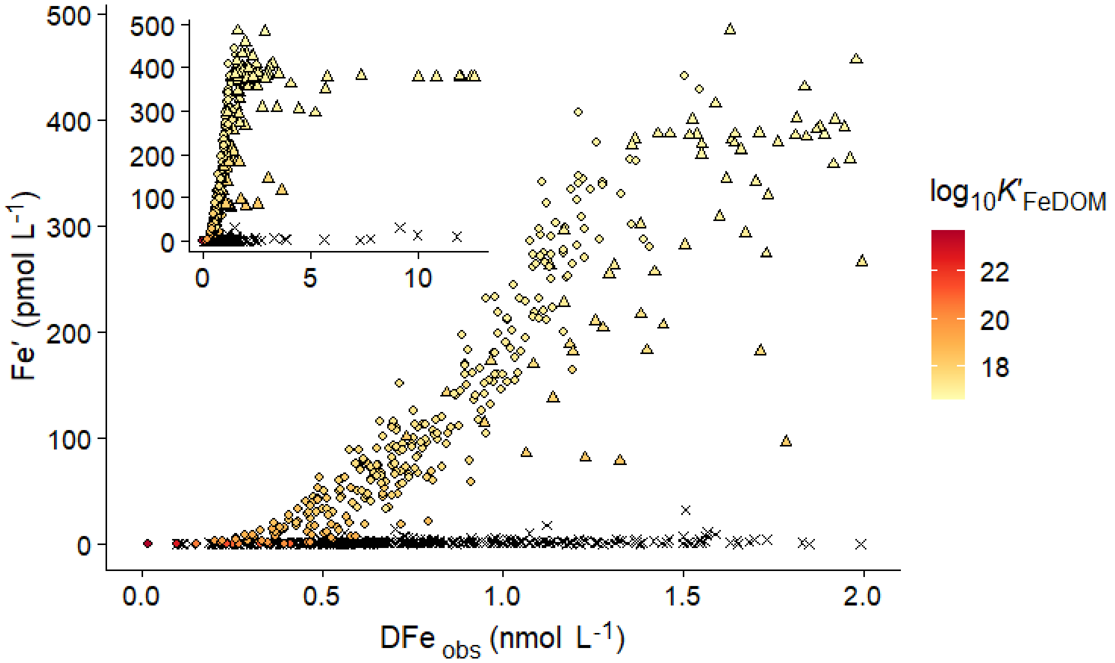

Supplementary Fig. 5. The impact of binding site heterogeneity on Fe′. Parameterisation of binding site heterogeneity results in a pronounced increase in Fe′ as DFe increases (open symbols). The decrease in the mean affinity of occupied binding sites allows for more precise determination of the Fe′ concentration at which authFeOH precipitates (triangles indicate data points where authFeOH is predicted). When Fe′ is calculated from a mean conditional stability constant and a total ligand concentration, a uniform Fe affinity for binding sites is assumed, which overestimates the amount of binding sites that can compete with authFeOH formation. For example, Fe′ calculated from conditional stability constants and ligand concentrations (black crosses) observed across the SPO at 16°S[16] using equations 2-9 (Supplementary Methods) does not increase sufficiently to exceed its saturation state, hence no authFeOH is predicted to precipitate. Inorganic Fe is not saturated even in close proximity to the Peruvian Shelf and at a prominent vent site at the EPR where DFe concentrations are higher than 10 nmol L^{-1} (see inset) and authFeOH phases were observed in particles[21].

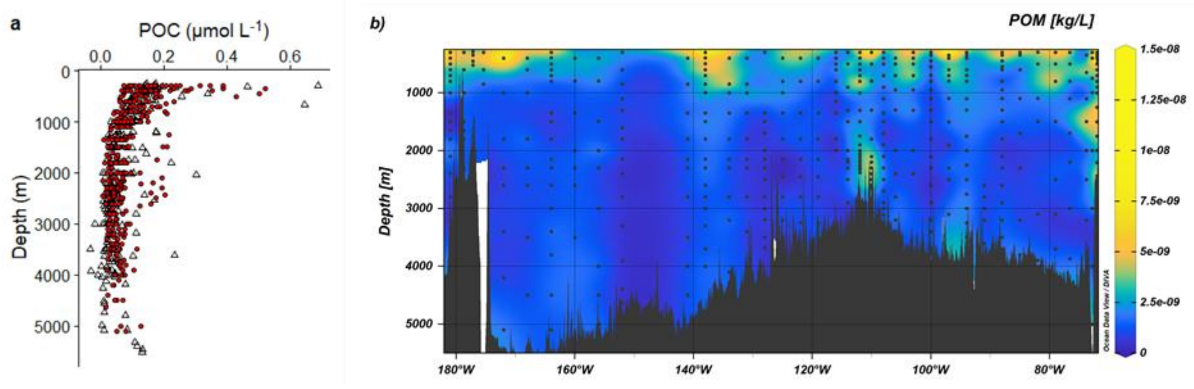

Supplementary Fig. 6. Estimation of amount of particulate organic matter across the SPO. a Estimates of particulate organic carbon (POC) calculated (red circles) from TPP concentrations observed on the GP21 transect using a C:P ratio of 80[22–24]. White tringles show the POC concentrations observed on GEOTRACES cruise GP16 at 16°S[22,25]. **b** Particulate organic matter (POM; note non-linear colour bar) used in our calculations. Black points show the positions of each calculation.

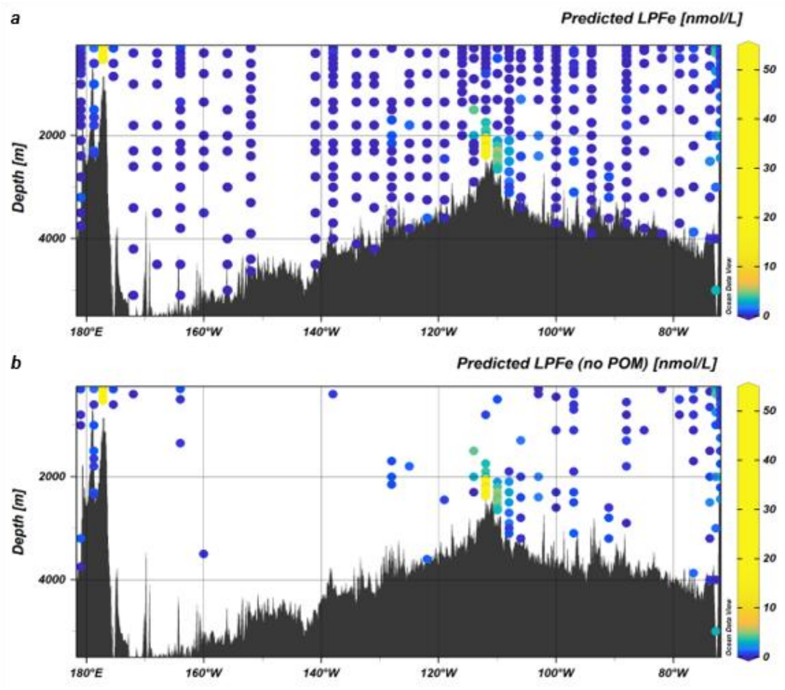

Supplementary Fig. 7a Predicted LPFe from pathway #1 to #4 shown as coloured data points (the data shown here underpin the average weighted gridded data shown in Fig. 7b). **b** Calculations that used TLFe as the total Fe input, but omitted POM (Supplementary Fig. 2f) result in complete absence of predicted LPFe across much of the SPO (265 out of 403 data points).

References

1. Meeussen, J. C. L. Orchestra: An object-oriented framework for implementing chemical equilibrium models. *Environmental Science and Technology* **37**, 1175–1182 (2003).
2. Millero, F. J. *Chemical Oceanography*. (CRC Press).
3. Eary, L. E. & Jenne, E. A. *Version 4. 00 of the MINTEQ Geochemical Code*. https://www.osti.gov/biblio/7073252 (1992) doi:10.2172/7073252.
4. Kinniburgh, D. G. *et al.* Ion binding to natural organic matter: competition, heterogeneity, stoichiometry and thermodynamic consistency. *Colloids and Surfaces A: Physicochemical and Engineering Aspects* **151**, 147–166 (1999).
5. Koopal, L., Tan, W. & Avena, M. Equilibrium mono- and multicomponent adsorption models: From homogeneous ideal to heterogeneous non-ideal binding. *Advances in colloid and interface science* **280**, (2020).

6. Langmuir, I. The constitution and fundamental properties of solids and liquids. Part 2. Liquids. *Journal of the American Chemical Society* **39**, 1848–1906 (1917).
7. Gerringa, L. J. A., Herman, P. M. J. & Poortvliet, T. C. W. Comparison of the linear van den Berg/Ruzic transformation and a non-linear fit of the Langmuir isotherm applied to Cu speciation data in the estuarine environment. *Marine Chemistry* **48**, 131–142 (1995).
8. Perdue, E. M. & Lytle, C. R. Distribution Model for Binding of Protons and Metal Ions by Humic Substances. *Environmental Science and Technology* **17**, 654–660 (1983).
9. Benedetti, M. F., van Riemsdijk, W. H. & Koopal, L. K. Humic Substances Considered as a Heterogeneous Donnan Gel Phase. *Enviromental Science and Technology* **30**, 1805–1813 (1996).
10. Lodeiro, P., Rey-Castro, C., David, C., Humphreys, M. P. & Gledhill, M. Proton binding characteristics of dissolved organic matter extracted from the North Atlantic. *Environmental Science & Technology* **57**, 21136–21144 (2023).
11. Milne, C. J., Kinniburgh, D. G., van Riemsdijk, W. H. & Tipping, E. Generic NICA−Donnan model parameters for metal-ion binding by humic substances. *Environmental Science & Technology* **37**, 958–971 (2003).
12. David, C. *et al.* Competition effects in cation binding to humic acid: Conditional affinity spectra for fixed total metal concentration conditions. *Geochimica et Cosmochimica Acta* **74**, 5216–5227 (2010).
13. Gerringa, L. J. A., Rijkenberg, M. J. A., Thuróczy, C.-E. & Maas, L. R. M. A critical look at the calculation of the binding characteristics and concentration of iron complexing ligands in seawater with suggested improvements. *Environmental Chemistry* **11**, 114–136 (2014).
14. Pižeta, I. *et al.* Interpretation of complexometric titration data: An intercomparison of methods for estimating models of trace metal complexation by natural organic ligands. *Marine Chemistry* **173**, 3–24 (2015).

15. Gledhill, M. & Gerringa, L. J. A. The effect of metal concentration on the parameters derived from complexometric titrations of trace elements in seawater—a model study. *Frontiers in Marine Science* **4**, 254 (2017).
16. Buck, K. N., Sedwick, P. N., Sohst, B. & Carlson, C. A. Organic complexation of iron in the eastern tropical South Pacific: Results from US GEOTRACES Eastern Pacific Zonal Transect (GEOTRACES cruise GP16). *Marine Chemistry* **201**, 229–241 (2018).
17. Pižeta, I. & Branica, M. Simulation and fitting of anodic stripping voltammetry data for determination of the metal complexing capacity. *Analytica Chimica Acta* **351**, 73–82 (1997).
18. Hudson, R. J. M., Rue, E. L. & Bruland, K. W. Modeling complexometric titrations of natural water samples. *Environmental Science & Technology* **37**, 1553–1562 (2003).
19. Lauvset, S. K. *et al.* GLODAPv2.2022: the latest version of the global interior ocean biogeochemical data product. *Earth System Science Data* **14**, 5543–5572 (2022).
20. Gledhill, M., Zhu, K., Rusiecka, D. & Achterberg, E. P. Competitive interactions between microbial siderophores and humic-like binding sites in European Shelf Sea waters. *Frontiers in Marine Science* **9**, 595 (2022).
21. Fitzsimmons, J. N. *et al.* Iron persistence in a distal hydrothermal plume supported by dissolved-particulate exchange. *Nature Geoscience* **10**, 195–201 (2017).
22. Pavia, F. J. *et al.* Shallow particulate organic carbon regeneration in the South Pacific Ocean. *Proceedings of the National Academy of Sciences* **116**, 9753–9758 (2019).
23. Lee, J.-M., Heller, M. I. & Lam, P. J. Size distribution of particulate trace elements in the U.S. GEOTRACES Eastern Pacific Zonal Transect (GP16). *Marine Chemistry* **201**, 108–123 (2018).
24. GEOTRACES Intermediate Data Product Group. The GEOTRACES Intermediate Data Product 2021. *NERC EDS British Oceanographic Data Centre* (2021).
25. Subhas, A. V., Pavia, F. J., Dong, S. & Lam, P. J. Global trends in the distribution of biogenic minerals in the ocean. *Journal of Geophysical Research: Oceans* **128**, e2022JC019470 (2023).
